# Hexagonal MoTe_2_ with Amorphous BN Passivation Layer for Improved Oxidation Resistance and Endurance of 2D Field Effect Transistors

**DOI:** 10.1038/s41598-018-26751-4

**Published:** 2018-06-06

**Authors:** Benjamin Sirota, Nicholas Glavin, Sergiy Krylyuk, Albert V. Davydov, Andrey A. Voevodin

**Affiliations:** 1 Department of Materials Science and Engineering, Advanced Materials and Manufacturing Processing Institute, University of North Texas, Denton, TX 76203 USA; 2 Materials and Manufacturing Directorate, Air Force Research Laboratory, Wright-Patterson AFB, OH 45433 USA; 3 Theiss Research, Inc, La Jolla, CA 92037 USA; 4 Materials Science and Engineering Division, National Institute of Standards and Technology, Gaithersburg, MD 20899 USA

## Abstract

Environmental and thermal stability of two-dimensional (2D) transition metal dichalcogenides (TMDs) remains a fundamental challenge towards enabling robust electronic devices. Few-layer 2H-MoTe_2_ with an amorphous boron nitride (a-BN) covering layer was synthesized as a channel for back-gated field effect transistors (FET) and compared to uncovered MoTe_2_. A systematic approach was taken to understand the effects of heat treatment in air on the performance of FET devices. Atmospheric oxygen was shown to negatively affect uncoated MoTe_2_ devices while BN-covered FETs showed considerably enhanced chemical and electronic characteristic stability. Uncapped MoTe_2_ FET devices, which were heated in air for one minute, showed a polarity switch from n- to p-type at 150 °C, while BN-MoTe_2_ devices switched only after 200 °C of heat treatment. Time-dependent experiments at 100 °C showed that uncapped MoTe_2_ samples exhibited the polarity switch after 15 min of heat treatment while the BN-capped device maintained its n-type conductivity for the maximum 60 min duration of the experiment. X-ray photoelectron spectroscopy (XPS) analysis suggests that oxygen incorporation into MoTe_2_ was the primary doping mechanism for the polarity switch. This work demonstrates the effectiveness of an a-BN capping layer in preserving few-layer MoTe_2_ material quality and controlling its conductivity type at elevated temperatures in an atmospheric environment.

## Introduction

Recent advances in the field of 2D materials have focused on molecular-thin, TMDs as semiconductor counterparts to graphene in the use of nanoscale electronics^1–5^. One such TMD is MoTe_2_, which offers a unique combination of a semiconductor (hexagonal 2 H polytype) to metal (trigonal 1 T′ polytype) phase transition, indirect-to-direct bandgap transition in low-dimensional limits, spin-orbit coupling, strong photoluminescence response, and high transistor on/off ratios^6–9^. Monolayer 2H-MoTe_2_ has a direct band gap of 1.10 eV, which transitions to an indirect band gap of 0.81 eV for bulk MoTe_2_ as the layer number increases^10^. The charge carrier quantum confinement, high mobility, and reduced interaction with adjusted layers due to a lack of dangling surface bonds has initiated an extensive number of studies regarding monolayer TMDs for electrical and optoelectronic devices. In this respect, field effect transistors made of 2D MoTe_2_ have been demonstrated to exhibit p-type^11,12^, n-type^13^, and ambipolar^14^ behavior. MoTe_2_ FETs have been reported to have on/off ratios above 10^6^, subthreshold swing of 140 mV/dec, and Hall mobilities around 10 cm^2^ (V s)^−1^ ^11^. Such properties with an interesting ability for ambipolar behavior suggest monolayer 2H-MoTe_2_ can be attractive for semiconducting devices as compared to other 2D TMDs.

A major hindrance of many 2D TMDs is their limited stability in air, especially when they are subjected to moderate heating during processing or device operation. While the perfect TMD monolayers are relatively stable, the presence of defects, such as point defects and edges of single crystal domains, play a decisive role in determining the stability of 2D TMDs when exposed to an oxidizing environment. Oxygen substitution of chalcogen atoms and oxidation along the crystal edges are the most commonly reported culprits for the deterioration of charge confinement and transport characteristics of 2D TMDs^15,16^. From this perspective, the packaging of 2D TMDs layers within electrically insulating and dielectric (for FET applications) thin layers, which can also prevent oxidation and deterioration of 2D TMD characteristics, is of a significant practical interest to advance 2D TMDs for device applications. The challenge of such passivation layers is in avoiding interaction of the 2D TMD layers with the adjusted electrically insulating materials, where typical thin dielectrics are mostly based on oxides (e. g., SiO_2_, Al_2_O_3_, HfO_2_), which intrinsically bring oxygen atoms in contact with TMDs and can contribute to TMD deterioration. This becomes especially prevalent if elevated temperatures are encountered during dielectric layer deposition or device operation. Thus, an exploration of a non-oxide based and environmentally stable dielectric material to be used in conjunction with TMD FET devices, or other 2D materials such as graphene and black phosphorus, and would benefit the wider usage of such materials.

Boron nitride has emerged as a dielectric counterpart to TMDs due to its chemical stability, lack of dangling bonds, and wide bandgap (5.97 eV) characteristics^17^. Most notably, hexagonal boron nitride (h-BN), which has a similar crystal structure to graphene^18^, can be exfoliated onto various 2D materials to form heterostructures such as h-BN-MoS_2_^19–21^, h-BN-WS_2_^22^, h-BN-MoSe_2_^23^, and BN-WSe_2_-BN^24^ with remarkable electrical properties^25^. While these studies clearly demonstrate the benefit of h-BN packaging with TMD for improved mobility, on/off ratio, and thermal management, the challenge of exfoliation routes for such 2D heterostructure device assemblies is prohibitive for the scalable and cost-effective device production, leading to the search of alternative routes for h-BN synthesis and integration with TMD structures^18^. Deposition of h-BN layers via chemical vapor deposition routes have been extensively explored but require high temperatures during synthesis and annealing^26–29^, reducing applicability for temperature sensitive TMDs.

Most recently, research involved in physical vapor deposition (PVD) of BN thin films has demonstrated the advantage of room temperature growth of amorphous BN (a-BN) layers on the order of 2 nm to 3 nm thickness over large areas, which show a bandgap of 4.5 eV, dielectric constant of about 6 and breakdown voltage on the order of 10 MV/cm^30,31^. This makes them comparable to exfoliated h-BN for dielectric applications. Passivation layers of PVD-grown a-BN in combination with graphene^32^, 2D WS_2_ and MoS_2_^33^, have shown improved field effect mobility and reduced background charge carrier concentration. Herein, we investigate devices composed of exfoliated 2 H MoTe_2_ with a PVD-grown a-BN passivation layer to study chemical and structural stability of 2D MoTe_2_ at elevated temperatures. The studies also include an analysis of FET device performance (including n- to p-type behavior transition, on/off ratio, and carrier mobility) after heating in air, covering a possible range of temperatures of such 2H-MoTe_2_/a-BN device applications in electronic devices. Formation of Te vacancy sites and the potential for atmospheric oxygen to form substitutional dopants were of critical attention in the performed study. We find this BN capping layer to be an effective barrier layer, which protects the 2D TMD material from detrimental chemical oxidation, and significantly reduces the rate of field effect mobility and on/off ratio degradation of FET devices as compared to the uncapped sample.

## Results and Discussion

### Material exfoliated and characterization

**Figure 1:**
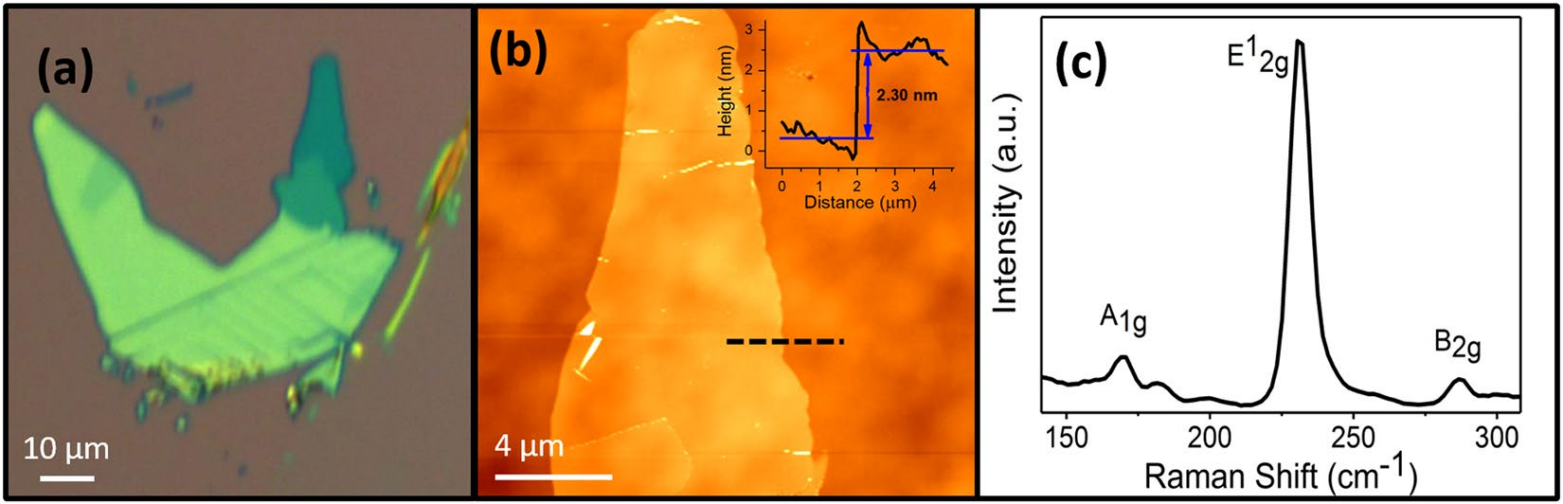
(**a**) An optical image of an MoTe_2_ exfoliated flake on SiO_2_/Si substrate: the few-layer section can be identified by the dark green color while the bulk is a lighter blue-green. (**b**) AFM image and line scan (dashed black line) from the few-layer section of flake in 1a: the height profile from the line scan measures a difference of 2.30 nm, which corresponds to 3 monolayers of MoTe_2_. (**c**) The Raman spectrum from MoTe_2_ flake, which is in agreement with few-layer MoTe_2_.

Figure 1a depicts an optical image of an exfoliated 2H-MoTe_2_ flake. The optical contrast of few layers and bulk flakes on SiO_2_/Si substrates provides a good guide to the flake thickness, since it is known that 2D flakes that contain one to few layers show a darker optical color than bulk material due to altering optical contrast with the SiO_2_/Si substrate^34^. AFM from a non-contact scan is shown in Fig. 1b, demonstrating good thickness uniformity of the dark-green area from Fig. 1a. A line scan measures a height difference of 2.30 nm, which corresponds to tri-layer MoTe_2_^35^. The Raman spectrum in Fig. 1c, clearly shows three distinct peaks typical for MoTe_2_ at 170 cm^−1^, 235 cm^−1^ and 289 cm^−1^, corresponding to the A_1g_, E^1^_2g_ and B_2g_ phonon modes, respectively^36^. The A_1g_ mode is due to out-of-plane phonon vibrations and is typically weak for 532 nm laser excitations^37^. The E^1^_2g_ peak is the dominant mode and corresponds to in-plane atomic oscillations, and it has been shown to exhibit a strong signal for MoTe_2_ in the 2D form and is relatively weak for bulk material^36^. The B_2g_ mode corresponds to the out-of-plane interaction of adjacent layers and has an interesting property in which it is clearly present for 2 to 5 of MoTe_2_ layers but absent for either monolayer or the bulk thickness. This peak was reported to have its strongest value for 2 layers and rapidly decreasing as the thickness increases until it disappears after 5 layers^34^. Therefore, the relative intensities of the E^1^_2g_ and B_2g_ peaks can be used to determine layer thickness and verify AFM measurements as presented in Fig. 1b. For all MoTe_2_ flakes selected for this study, the Raman spectra displayed a strong E^1^_2g_ peak and less intense A_1g_ and B_2g_ peaks, which is in agreement with 3-layer thickness measurements with AFM (Fig. 1c).

**Figure 2:**
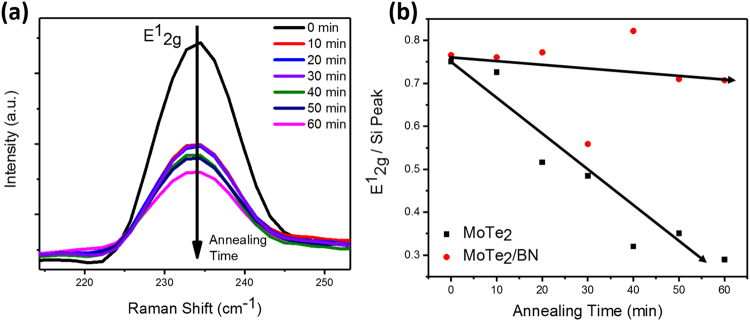
(**a**) Raman spectra of the E^1^_2g_ peak intensity variation for few monolayers thick 2H-MoTe_2_ flakes exposed to 100 °C in air for the shown time intervals. (**b**) Comparison of E^1^_2g_ peak intensities changes as a function of 100 °C in air holding time for uncapped MoTe_2_ and BN-capped MoTe_2_ flakes; to facilitate the comparisons and avoid errors due to absolute peak intensity changes over time, the peak intensity was normalized to the Si substrate peak (520.5 cm^−1^). The shown linear fitting was used to calculate E^1^_2g_ peak intensity decline due to oxidation processes for 2H-MoTe_2_ and 2H-MoTe_2_/a-BN.

In order to analyze the 2D MoTe_2_ thermal degradation rate over time, Raman spectra comparing the 2H-MoTe_2_ E^1^_2g_ mode for samples heated at 100 °C for up to 60 min with 10 min increment were collected and are shown in Fig. 2a. Gauss peak fitting of the E^1^_2g_ mode had determined that this vibration mode maintains its location at ≈234 cm^−1^ throughout the experiment, which suggests the 2H-MoTe_2_ did not undergo a thickness change after 60 min of 100 °C heat treatment in air. However, the E^1^_2g_ peak exhibits a continuous intensity decline from its max value at room temperature, and the rate of this decline is strongly affected by the presence of the BN cap layer. The Raman analysis did not show the presence of MoO_3_ or MoO_2_ vibration modes for both uncapped and a-BN capped 2H-MoTe_2_ flakes, suggesting that no individual phases of molybdenum oxide crystal structure was formed. However, XPS analysis discussed below shows that heating in air had resulted in surface oxidation. Normalizing the E^1^_2g_ peaks to the Raman peak of silicon at 520 cm^−1^ allows for comparison between uncapped and BN capped flakes. Figure 2b clearly shows that the uncapped MoTe_2_ exhibits a much greater rate of decay of the E^1^_2g_ Raman mode. Such is likely brought by damping the inter-layer oscillation modes with increased lattice imperfections and internal atomic stresses from Te vacancies and oxygen substitutions. In contrast, normalized Raman spectra of the BN-capped MoTe_2_ sample maintained its E^1^_2g_ peak intensity after 60 min of heating, see Supplementary data Figure S1. Applying a linear fit to the data, the slopes of the fitting lines suggest that the decay rate for the E^1^_2g_ mode in 2H-MoTe_2_ material with 100 °C exposure time is an order of magnitude higher than that for the 2H-MoTe_2_/a-BN, (8.3 × 10^−3^ min^−1^ versus 8.1 × 10^−4^ min^−1^, respectively). The results clearly demonstrated the benefit of the BN capping layer for the preservation of 2H-MoTe_2_ structure at elevated temperatures in air.

**Figure 3:**
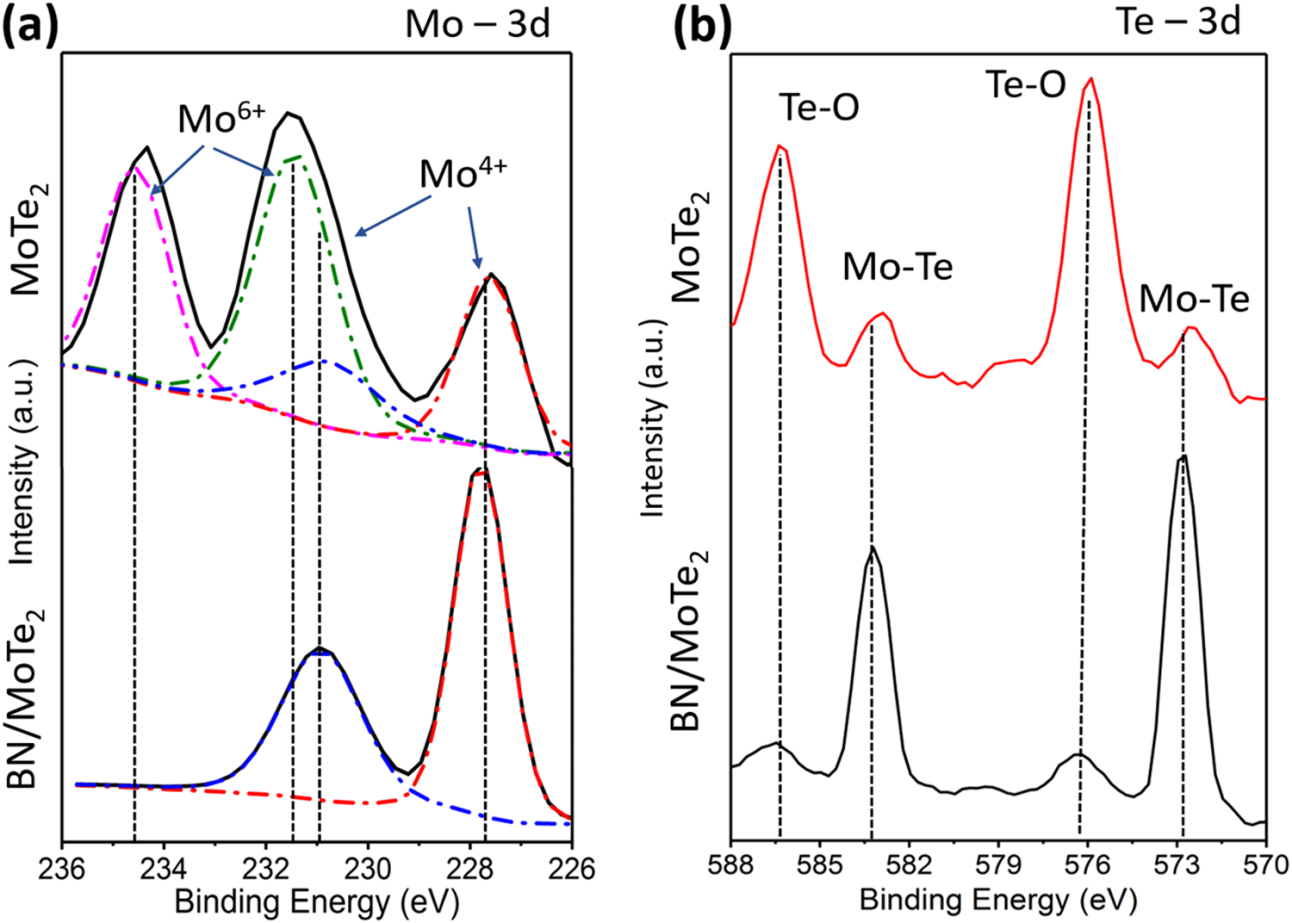
XPS analysis of the (**a**) Mo 3d and (**b**) Te 3d binding energies for 2H-MoTe_2_ and 2H-MoTe_2_/a-BN flakes after 60 minutes of 100 °C heating. The BN-capped sample maintains its Mo-Te bonding, demonstrating increased resistance to oxidation, while the appearance of Mo-O and Te-O bonds in the uncapped device suggests significant oxidation of MoTe_2_ to form MoO_3_ and TeO_2_ phases.

In order to investigate in more detail, the chemical state changes of MoTe_2_ after exposure to elevated temperatures in air, XPS analysis was conducted on exfoliated flakes with and without BN layers after heating at 100 °C for 60 min. The results are presented in Fig. 3, and XPS spectra of MoTe_2_ flakes before heating can be found in Supplemental Figure S2. The uncoated MoTe_2_ flake shows three distinct peaks for the Mo 3d orbital, which can be seen in the top spectrum of Fig. 3a. The recorded spectra deconvolutions with 3d doublets reveal a clear presence of two chemical states with Mo 3d_3/2_ peak locations at 234.6 eV and 231.5 eV. The higher binding energy doublet belongs to Mo^6+^ in MoO_3_ bonding, and the lower one to Mo^4+^ in MoTe_2_^38^. This suggests partial oxidation of the MoTe_2_ surface. In a sharp contrast, similar analysis of the MoTe_2_/BN sample after 60 min exposure to 100 °C in air shows only one doublet with Mo 3d_3/2_ peak location at 231.4 eV, indicating the absence of molybdenum oxidation when the BN capping layer was applied. Similar observation of the BN-capping layer preventing oxidation can be made from corresponding XPS spectra of Te 3d, shown in Fig. 3b. The un-capped MoTe_2_ (upper spectrum) has dominant Te-O peaks at 575.92 eV and 586.42 eV, corresponding to TeO_2_ formation. On the other hand, the BN-capped (lower spectrum) sample has dominant peaks at 572.9 eV and 583.4 eV and significantly weaker Te-O peaks at 576.3 eV and 586.8 eV. This suggests that heating of the uncapped MoTe_2_ in air results in possible oxidation as evidenced by the appearance of Mo-O and Te-O bonds in the XPS spectra. This process was significantly suppressed for the MoTe_2_/BN sample. The XPS analysis corroborate the Raman studies that the application of the 10 nm thin BN layer can effectively resist oxidation of MoTe_2_ at elevated temperatures in air.

### Electrical Measurements of Uncapped and BN-capped devices

**Figure 4:**
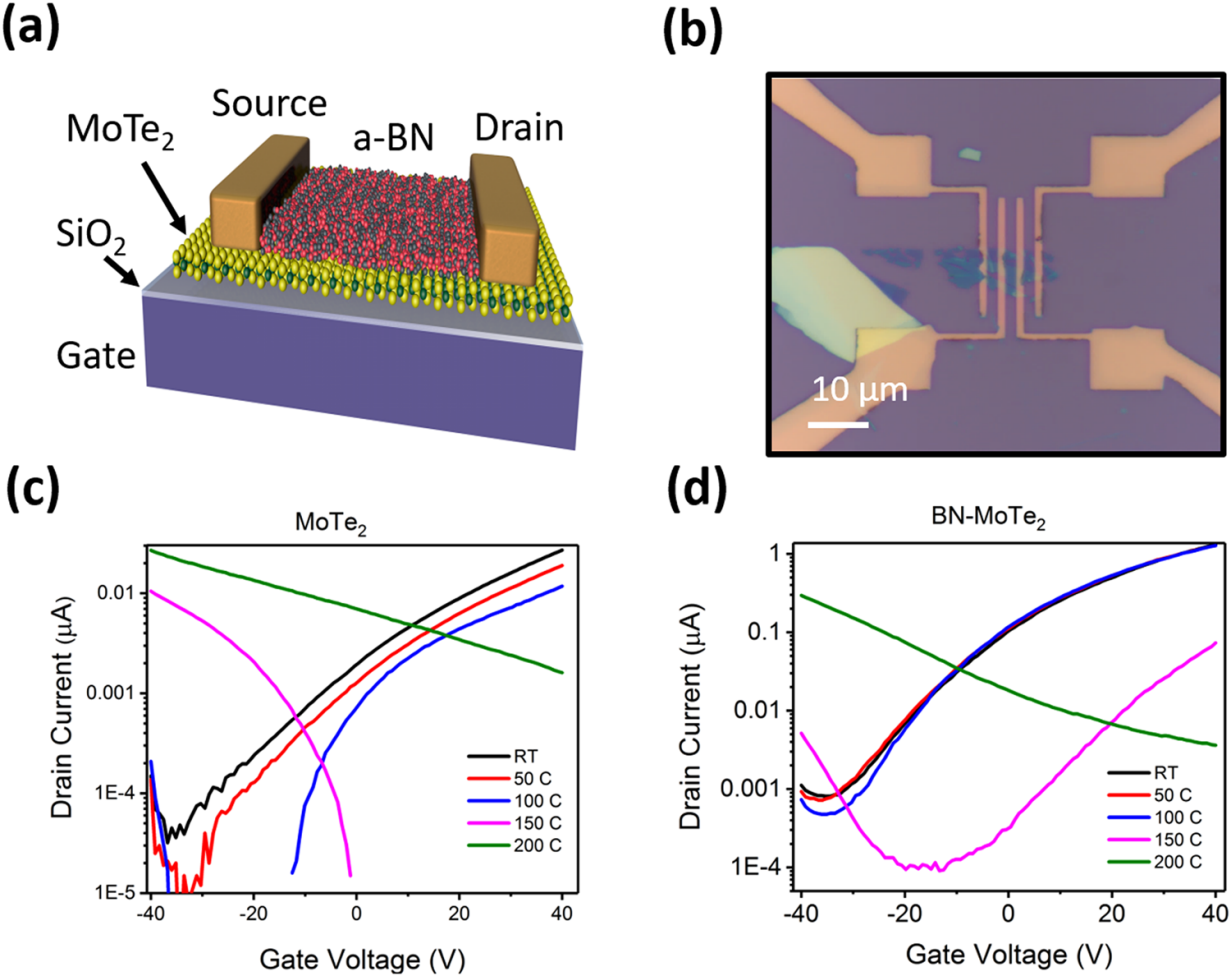
(**a**) Schematic cross-section of FET back-gated device with BN capped MoTe_2_. (**b**) Optical image of typical MoTe_2_ few-layer flake with Ti/Au source drain contacts deposited by EBL. (**c**) Examples of drain current measurements as a function of gate voltage for a backdated MoTe_2_ device. The drain voltage was maintained at 1 VDC. Uncovered MoTe_2_ device shows initial n-type semiconducting behavior that switches to p-type after 150 °C. (**d**) The BN/MoTe_2_ device maintains a much greater degree of stability through 100 °C and exhibits a polarity switch to p-type behavior much later at 200 °C.

To determine the effect of an a-BN capping layer on the retaining charge transport characteristics and MoTe_2_ based FET device performance upon exposure to elevated temperature in air back-gated FET devices were fabricated from few-layer thick MoTe_2_ flakes on SiO_2_ using electron beam lithography. This amorphous BN layer was previously determined to be pin-hole free dielectric material with breakdown characteristic and dielectric constant exceeding CVD grown h-BN films, approaching that of mechanically exfoliated h-BN^30,31^. Such deposition method is ideal for a cost-effective application of dielectric BN layer for 2D heterostructures as compared with other 2D BN growth methods^26^. In the recent studies of PLD grown amorphous BN it was found that the dielectric properties were close to that of h-BN and be as smooth as underlying substrates, where graphene, MoS_2_, WS_2_ and a variety of metal and ceramic substrates were tested with an equal result in a-BN smoothness and pin-hole free morphology over several cm^2^ areas^30,31,39^. While there were no systematic studies to determine a-BN inertness, the earlier explorations with a-BN/graphene heterostructures had shown a significant increase of mobility in graphene monolayers when it was sandwiched with a-BN, relating this to a-BN inertness, similar to monolayer thin h-BN^32^. Stabilization of charge transport characteristics of epitaxially grown graphene in air by the application of a capping a-BN layer was also reported^40,41^. We then reasonably expected a similar effect with MoTe_2_ devices, and such is explored for the first time in this study at elevated temperatures in air. Back-gated FET device schematic made from MoTe_2_ and including a BN capping layer is depicted in Fig. 4a and an example of such fabricated device is shown in an optical image of Fig. 4b.

Electrical performance of back-gated FET devices annealed from room temperature to 300 °C is shown in Fig. 4. MoTe_2_/BN and MoTe_2_ as-exfoliated devices underwent the side by side elevated temperature in air exposure and were held for 1 min at the target temperature in atmospheric conditions before cooling down and measuring their performance. Figure 4c presents a semi-log plot of drain current (*I*_*dc*_) versus gate voltage (*V*_*g*_) for a MoTe_2_ device as the annealing temperature was systematically increased from room temperature to 300 °C at 50 °C increments. The device initially exhibits n-type behavior as indicated by the positive slope, with the maximum current decreasing slightly as the temperature is increased to 100 °C. At 150 °C (pink curve) the polarity switches to p-type behavior as indicated by the negative slope. At 200 °C, the device operates as a p-type but also exhibits semi-metallic behavior as indicated by the linear slope. For few-layer materials, this temperature is sufficient to sublime Te directly into the vapor state, leaving behind Mo atoms which would contribute to metallic properties.

A semi-log *I*_*dc*_ *- V*_*g*_ plot for a MoTe_2_/BN device is presented in Fig. 4d. The drain current curve remains very stable up to 100 °C. At 150 °C there is a sharp decline in current, however the device remains n-type, as indicated by the drain current curve slope for positive gate voltages. However, after heat treatment at 200 °C, the drain current switches to negative gate voltages and decreases for positive gate voltages, indicating a switch to p-type semiconductor operation. Once temperatures reached 250 °C we noticed the channel lost semiconductor properties and was unresponsive to gate voltage variation, which suggests degradation of MoTe_2_. At 300 °C, the Ti/Au contacts themselves failed and further temperature increase was not performed. Scanning electron microscopy studies of the contacts had shown dewetting of these thin metal films (Supplementary data Figure S3), resulting in a failure of conductive pathways. Such dewetting is expected for thin electrode materials due to the low melting point of gold, and experiments at higher temperatures may require other electrode materials.

Field effect mobility was calculated from the *I*_*dc*_ *- V*_*g*_ curves and is shown at various annealing temperatures in Table 1. Before performing any temperature exposure, the measurements of as fabricated devices (23 °C in Table 1) clearly indicate that the capping MoTe_2_ with BN has a significant effect on the mobility improvement as the measured values for MoTe_2_/BN devices were five times greater than that of uncapped MoTe_2_. The absolute values of the room temperature mobility measurements in the literature vary between 0.03 and 20 cm^2^V^−1^s^−1,^^11,12,14,42,43^. While our results are within this reported range, our study demonstrates the effect of a-BN capping layer on the device stability during heating as discussed below.

**Table 1:** Mobility of uncapped (MoTe_2_) and BN-capped (BN/MoTe_2_) devices as a function of temperature with carrier type.

	23 °C	50 °C	100 °C	150 °C	200 °C
MoTe_2_/BN	1.077, n-type	0.870, n-type	0.837, n-type	0.204, n-type	0.442, p-type
MoTe_2_	0.235, n-type	0.157, n-type	0.103, n-type	0.133, p-type	0.212, p-type

Values were calculated from data in Fig. 4. Mobility units are in cm^2^ (V s)^−1^.

The field-effect mobility of BN capped MoTe_2_ (red) is maintained around 0.8–1 cm^2^ (V s)^−1^ until 150 °C where it experiences a sharp drop to 0.2 cm^2^ (V s)^−1^. Furthermore, after 200 °C the mobility values are shown to switch polarity from n- to p-type. The un-coated MoTe_2_ in contrast, has a much lower initial mobility and undergoes its polarity switch already at 150 °C. Finally, at 200 °C the mobility absolute value increases slightly. The switch from initial n-type behavior to p-type can be linked to the oxygen incorporation in MoTe_2_ layers found from XPS studies (Fig. 3). Previous reports of DFT simulations of oxygen interactions with MoTe_2_ and formation of Mo-O and Te-O bonds can lead to the formation of deep-level trap states located 0.4 eV below the conduction band minimum^44^. Such states may act as acceptor sites, effectively resulting in a switch to the p-type behavior as the number of oxygen substitutes for tellurium in MoTe_2_ lattice is increased at higher temperatures in air. This is further supported by the delayed polarity switching of our experiments with BN covered device which protects MoTe_2_ from oxygen diffusion and subsequent substitution in Te sites.

**Figure 5:**
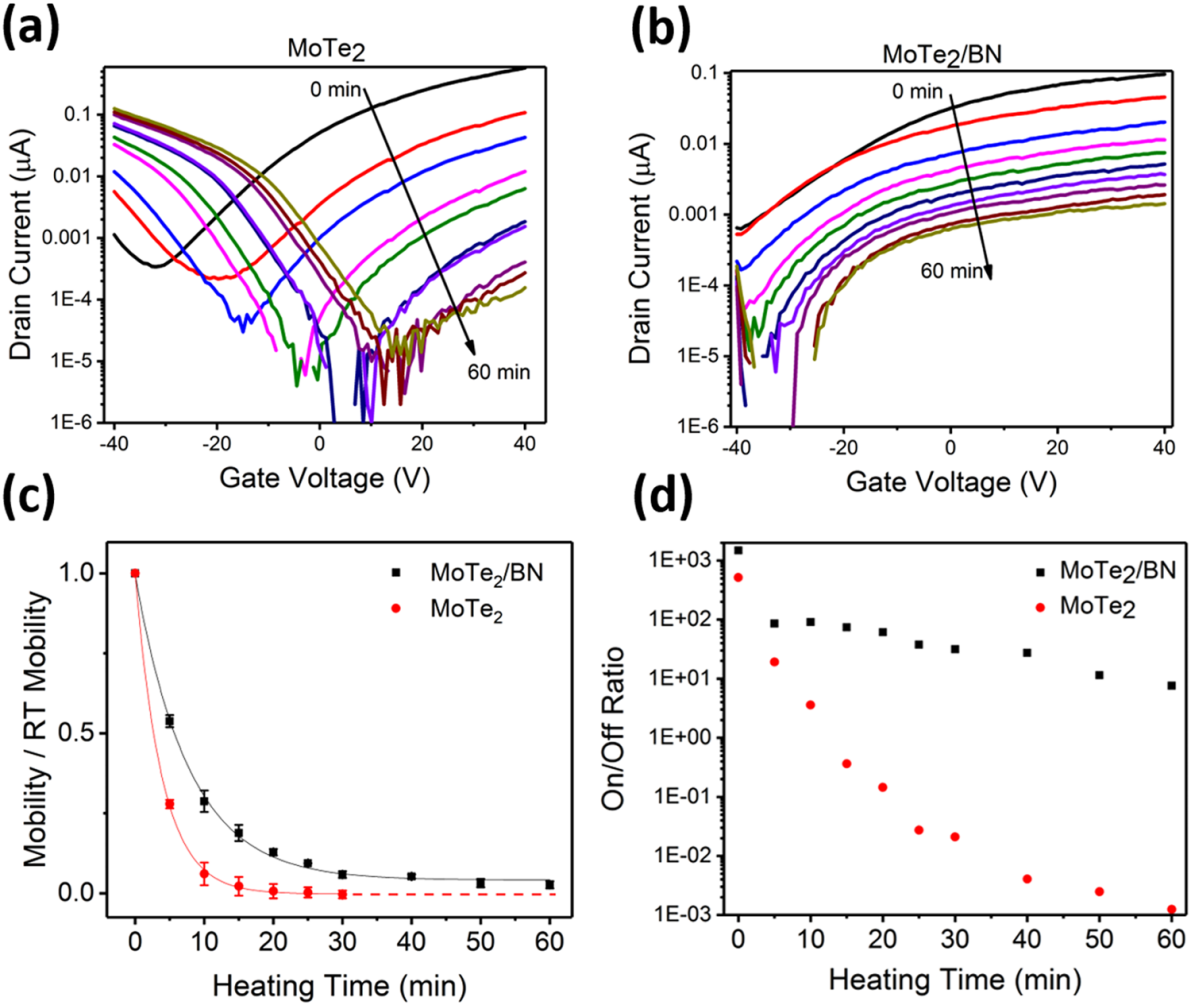
Current-voltage measurements of (**a**) MoTe_2_ and (**b**) BN/MoTe_2_ back-gated FET devices, which were held at 100 °C in air for consecutive time increments as shown in data labels. The drain voltage was maintained at 1 VDC. (**c**) Mobility of MoTe_2_ and MoTe_2_/BN devices, shown as a ratio to their initial mobility and plotted as a function of the time for device exposure to 100 °C in air. After 30 minutes, the electron mobility of the uncapped MoTe_2_ device had dropped below the measurement noise level and thus is not continued in the plot. (**d**) Semilog plot comparing on/off ratios of MoTe_2_ and MoTe_2_/BN devices. The BN-coated device maintains a significantly higher ratio throughout the experiment while the uncapped devices rapidly decreases.

To study the effects of the BN layer for improved endurance of MoTe_2_ device characteristics in oxidizing environments, the FET devices were held at a constant temperature of 100 °C for incrementally increased times from 5 min to up to one hour. *I*_*dc*_*-V*_*g*_ curves displaying the change in electrical properties of MoTe_2_ and MoTe_2_/BN over increased time of exposure to 100 °C in air are displayed as semi-log plots in Fig. 5a,b, respectively. The uncapped MoTe_2_ devices (Fig. 5a) exhibit a significant decrease in maximum drain current after the first 5 minutes, suggesting that the surface oxidation occurs almost immediately. Initially the device is active in the positive gate voltage region, indicating n-type behavior. However, as the heating time increases, the maximum drain currents decrease and transitions to the negative gate voltage region, indicating p-type behavior. Along this n- to p-type transition (about 10–15 min) the devices exhibit ambipolar behavior. For MoTe_2_/BN devices, (Fig. 5b), the maximum drain current also declines with greater heating times but more steadily and remains non-zero even after 60 minutes exposure to 100 °C in air. In addition, the MoTe_2_/BN devices do not undergo a polarity switch to p-type and never lose the n-type behavior (Fig. 5b).

In Fig. 5c, the field effect mobility determined after heating to 100 °C in air is plotted to investigate MoTe_2_ FET device endurance and the protective effect of the a-BN capping layer. The initial (before heating to 100 °C in air) values for electron-based mobility were used to normalize the data plot in order to demonstrate the rate of change of the device performance over time. Both MoTe_2_ and MoTe_2_/BN devices undergo a rapid mobility decline relative to their initial mobility after first 10 minutes before leveling off at 20 min to 60 min, suggesting that surface changes to MoTe_2_ occur very quickly. The uncapped MoTe_2_ devices show a larger decline in field effect mobility compared to MoTe_2_/BN devices. In addition, the MoTe_2_/BN devices retain their n-type performance over the entire test period without failing, confirming that the a-BN capping is a good choice for improving long-term device performance in addition to the improved overall mobility values discussed earlier. The on/off ratio change with exposure to 100 °C in air is presented in Fig. 5d. The MoTe_2_/BN device maintains a significantly higher ratio of around 10^2^ throughout the entire 60 min experiment time, while the on/off ratio for uncapped MoTe_2_ device rapidly decreases.

In conclusion, durability of future 2D TMD devices will rely on solutions to prevent material degradation during their fabrication and operation. Moderate heating of uncapped exfoliated 2H-MoTe_2_ in air shows a considerable oxidation of few-layer thick MoTe_2_ surface resulting in deterioration of the FET device performance. The oxidation can be substantially mitigated by capping the 2H-MoTe_2_ with a 10 nm thick a-BN layer, applied by pulsed laser deposition. For uncapped 2H-MoTe_2_, the temperature induced oxidation leads to MoTe_2_ structural defects with oxygen incorporation and appearance of Mo-O and Te-O bonding in XPS spectra. However, the absence of MoO_3_ structure in the Raman data signals that partial oxidation occurred in the form of oxygen defects in Te vacancy sites or physio-absorption to Te surface sites. This is accompanied by the reduction in the field effect mobility and an eventual switch from an n- to p-type semiconductor behavior at around 150 °C. Devices consisting of 2H-MoTe_2_ with an a-BN passivation layer were shown to have improved field effect mobility characteristics and significantly suppress material degradation via oxidation from heating in air. For fabricated MoTe_2_/BN FET devices, the oxidation induced polarity switch was delayed to a temperature of 200 °C and as such, retained their n-type mobility and stabilized on/off ratio in extended exposure to 100 °C in air. Hence, amorphous BN was found to be an effective approach for preventing 2H-MoTe_2_ oxidation and improving the 2D FET device endurance and overall performance. Considering a large area scalability, room temperature growth and versatility of the PLD produced a-BN passivation layer, the explored 2H-MoTe_2_/a-BN structures can be beneficial for electronic and optoelectronic device applications.

## Methods

### Preparation of 2H-MoTe_2_

2H-MoTe_2_ single-crystals were produced by the chlorine-assisted chemical vapor transport (CVT) method. In brief, a vacuum-sealed quartz ampoule with polycrystalline MoTe_2_ powder and a small amount of TeCl_4_ (4 mg/cm^3^) was placed in a furnace containing a temperature gradient so that the MoTe_2_ charge was kept at 800 °C, and the temperature at the opposite end of the ampoule was about 710 °C. The ampoule was slowly cooled after 7 days of growth. The 2 H phase of the obtained MoTe_2_ flakes was confirmed by powder X-ray diffraction and transmission electron microscopy studies.

### MoTe_2_ Device Fabrication

2H-MoTe_2_ flakes were then later mechanically exfoliated onto heavily p-doped silicon wafers with a 270 nm silicon oxide surface layer using conventional adhesive tape methods, the details of which have been reported previously^45^. Candidate 2D flakes were identified using optical microscopy since the SiO_2_/Si substrate provides a clear optical contrast for single, double, and few-layer flakes compared to bulk^10^. Flake thickness was estimated using Raman spectroscopy and further confirmed using atomic force microscopy (AFM). Back-gated FETs with channel widths of 2 µm were fabricated by evaporating Ti/Au (20 nm/20 nm) contacts onto the MoTe_2_ flakes defined by electron beam lithography (EBL) and lift-off.

### a-BN Deposition

Some of the devices were covered, post device fabrication, by an ultrathin amorphous BN layer. This a-BN capping layer of 10 nm thickness, was deposited by pulsed laser deposition using a BN target (99.999%), ablated with a focused 248 nm KrF laser in nitrogen background gas under processing parameters optimized for a fully dense, pin-hole free, and stoichiometric BN film growth as reported previously^30,39^. The a-BN capping layer growth covered the entire device and was deposited at room temperature. We did not notice an effect to the crystallinity of the MoTe_2_ or deposited Ti/Au electrodes.

### Electrical Measurements of Heated Devices

The electrical measurements were performed using an Agilent B2902A system equipped with a probe station. The 10 nm a-BN layer was completely pierced by the contact probes to make good metal contact with source and drains electrodes of the devices.

Heat treatment experiments were conducted using a hotplate in laboratory air of about 50% relative humidity. Two sets of heating experiments were performed: i) temperature dependent measurements where samples were heated from room temperature (≈23 °C) to 300 °C with a 50 °C increment and held for 1 min before cooling, and ii) time dependent measurements where samples were held at 100 °C with 5-min time increments up to 60 min before cooling down. Back-gated FET devices with both open and a-BN-capped MoTe_2_ surfaces were heated simultaneously to ensure experimental consistency. Before taking I-V characteristics, the devices were allowed to cool to room temperature to prevent thermal effects on the measured results and allow direct comparisons of device performances after heat treatment.

## Electronic supplementary material

Supplementary Information

## References

[1] Geim AK, Grigorieva IV (2013). Van der Waals heterostructures. Nature.

[2] Akinwande D, Petrone N, Hone J (2014). Two-dimensional flexible nanoelectronics. Nat Commun.

[3] Fiori G (2014). Electronics based on two-dimensional materials. Nature nanotechnology..

[4] Ganatra R, Zhang Q (2014). Few-layer MoS2: a promising layered semiconductor. ACS nano..

[5] Bhimanapati GR (2015). Recent advances in two-dimensional materials beyond graphene. ACS Nano..

[6] Lezama IG (2015). Indirect-to-direct band gap crossover in few-layer MoTe(2). Nano Lett.

[7] Cho S (2015). Phase patterning for ohmic homojunction contact in MoTe2. Science.

[8] Qi Y (2016). Superconductivity in Weyl semimetal candidate MoTe2. Nat Commun.

[9] Sankar R (2017). Polymorphic Layered MoTe2 from Semiconductor, Topological Insulator, to Weyl Semimetal. Chemistry of Materials.

[10] Keum DH (2015). Bandgap opening in few-layered monoclinic MoTe2. Nature Physics.

[11] Pradhan NR (2014). Field-effect transistors based on few-layered α-MoTe2. ACS Nano..

[12] Fathipour, S. *et al*. Exfoliated multilayer MoTe2 field-effect transistors. *Applied Physics Letters* **105**(19) (2014).

[13] Ji H (2016). Suppression of Interfacial Current Fluctuation in MoTe2 Transistors with DifferentDielectrics. ACS Appl. Mater. Interfaces.

[14] Nakaharai S, Yamamoto M, Ueno K, Tsukagoshi K (2016). Carrier Polarity Control in alpha-MoTe2 Schottky Junctions Based on Weak Fermi-Level Pinning. ACS Appl Mater Interfaces.

[15] Zhou H (2013). Thickness-dependent patterning of MoS2 sheets with well-oriented triangular pits by heating in air. Nano Research..

[16] Nan H (2014). Strong photoluminescence enhancement of MoS2 through defect engineering and oxygen bonding. ACS Nano..

[17] Pakdel A, Bando Y, Golberg D (2014). Nano boron nitride flatland. Chem Soc Rev.

[18] Dean CR (2010). Boron nitride substrates for high-quality graphene electronics. Nature nanotechnology..

[19] Lee GH (2013). Flexible and transparent MoS2 field-effect transistors on hexagonal boron nitride-graphene heterostructures. ACS nano..

[20] Wang S, Wang X, Warner JH (2015). All chemical vapor deposition growth of MoS2: h-BN vertical van der Waals heterostructures. ACS nano..

[21] Yan A (2015). Direct Growth of Single- and Few-Layer MoS2 on h-BN with Preferred Relative Rotation Angles. Nano Lett.

[22] Iqbal MW (2015). High-mobility and air-stable single-layer WS2 field-effect transistors sandwiched between chemical vapor deposition-grown hexagonal BN films. Sci Rep.

[23] Zhang Q (2016). Bandgap renormalization and work function tuning in MoSe2/hBN/Ru(0001) heterostructures. Nat Commun.

[24] Xu, S. *et al*. High-quality BN/WSe2/BN heterostructure and its quantum oscillations. Preprint at http://arxiv.org/abs/1503.08427 (2015).

[25] Bhimanapati, G. R., Glavin, N. R. & Robinson, J. A. In *2D Materials* 1st ed, Vol 95 (eds Iacopi, F., Boeckl, J. J. & Jagadish, C.) Ch. 3 (Elsevier, 2016).

[26] Song L (2010). Large scale growth and characterization of atomic hexagonal boron nitride layers. Nano Lett.

[27] Ismach A (2012). Toward the controlled synthesis of hexagonal boron nitride films. Acs Nano..

[28] Shi Y (2010). Synthesis of few-layer hexagonal boron nitride thin film by chemical vapor deposition. Nano letters..

[29] Bresnehan MS (2013). Prospects of direct growth boron nitride films as substrates for graphene electronics. Journal of Materials Research..

[30] Glavin NR (2014). Synthesis of few-layer, large area hexagonal-boron nitride by pulsed laser deposition. Thin Solid Films.

[31] Glavin NR (2016). Amorphous Boron Nitride: A Universal, Ultrathin Dielectric For 2D Nanoelectronics. Advanced Functional Materials.

[32] Uddin, M. A. *et al*. Mobility enhancement in graphene transistors on low temperature pulsed laser deposited boron nitride. *Applied Physics Letters* **107**(20) (2015).

[33] McConney ME (2016). Direct synthesis of ultra-thin large area transition metal dichalcogenides and their heterostructures on stretchable polymer surfaces. Journal of Materials Research.

[34] Yamamoto (2014). Strong enhancement of Raman scattering from a bulk-inactive vibrational mode in few-layer MoTe2. ACS Nano..

[35] Puotinen D, Newnham RE (1961). The crystal structure of MoTe2. Acta Crystallographica..

[36] Goldstein T (2016). Raman scattering and anomalous Stokes-anti-Stokes ratio in MoTe2 atomic layers. Sci Rep..

[37] Ruppert C, Aslan OB, Heinz TF (2014). Optical properties and band gap of single- and few-layer MoTe2 crystals. Nano Lett..

[38] Vishwanath S (2018). MBE growth of few-layer 2H-MoTe2 on 3D substrates. Journal of Crystal Growth.

[39] Glavin NR (2015). Temporally and spatially resolved plasma spectroscopy in pulsed laser deposition of ultra-thin boron nitride films. Journal of Applied Physics.

[40] Rigosi AF (2017). Preservation of Surface Conductivity and Dielectric Loss Tangent in Large-Scale, Encapsulated Epitaxial Graphene Measured by Noncontact Microwave Cavity Perturbations. Small..

[41] Rigosi AF (2017). Electrical Stabilization of Surface Resistivity in Epitaxial Graphene Systems by Amorphous Boron Nitride Encapsulation. ACS Omega..

[42] Lin YF (2014). Ambipolar MoTe2 transistors and their applications in logic circuits. Adv. Mater..

[43] Huang H (2016). Highly sensitive visible to infrared MoTe2 photodetectors enhanced by the photogating effect. Nanotechnology.

[44] Chen B (2015). Environmental changes in MoTe2 excitonic dynamics by defects-activated molecular interaction. ACS Nano..

[45] Huang Y (2015). Reliable exfoliation of large-area high-quality flakes of graphene and other two-dimensional materials. ACS nano..

