# Supplementary material for: Hexagonal MoTe2 with Amorphous BN Passivation Layer for Improved Oxidation Resistance and Endurance of 2D Field Effect Transistors
Source: Sci Rep. 2018 Jun 6;8:8668. doi: 10.1038/s41598-018-26751-4 (PMC5989207; doi:10.1038/s41598-018-26751-4)
Supplement: Supplementary file 1 — Supplementary Information [file 41598_2018_26751_MOESM1_ESM.pdf]

Hexagonal $MoTe_2$ with Amorphous BN Passivation Layer for Improved Oxidation Resistance and Endurance of 2D Field Effect Transistors

Benjamin Sirota[1], Nicholas Glavin[2], Sergiy Krylyuk[3, 4], Albert V. Davydov[4] and Andrey A. Voevodin[1*]

[1] *Materials Science and Engineering, Advanced Materials and Manufacturing Processing Institute, University of North Texas, Denton, TX 76203, USA*

[2] *Materials and Manufacturing Directorate, Air Force Research Laboratory, Wright-Patterson AFB, OH 45433, USA*

[3] Theiss Research, Inc., La Jolla, CA 92037, USA.

[4] Materials Science and Engineering Division, National Institute of Standards and Technology, Gaithersburg, MD 20899, USA

* Correspondence to Andrey.Voevodin@unt.edu

Supplementary Information:

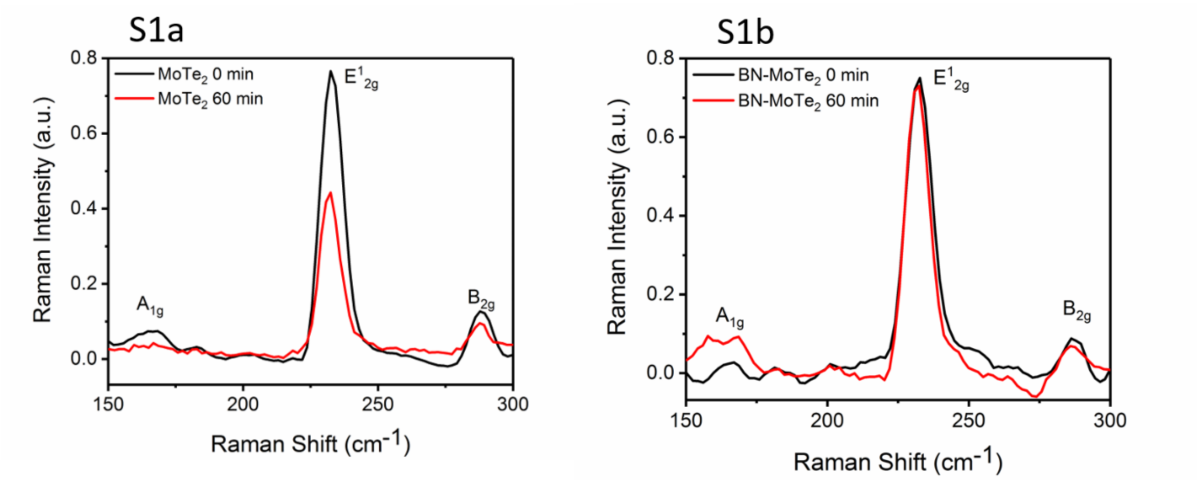

Supplementary Figure 1. Raman spectra of uncapped and BN-capped $MoTe_2$ before and after 60 min of heating at 100 °C. Data was normalized to the substrate's Si 520.5 cm^{-1} peak (not shown).

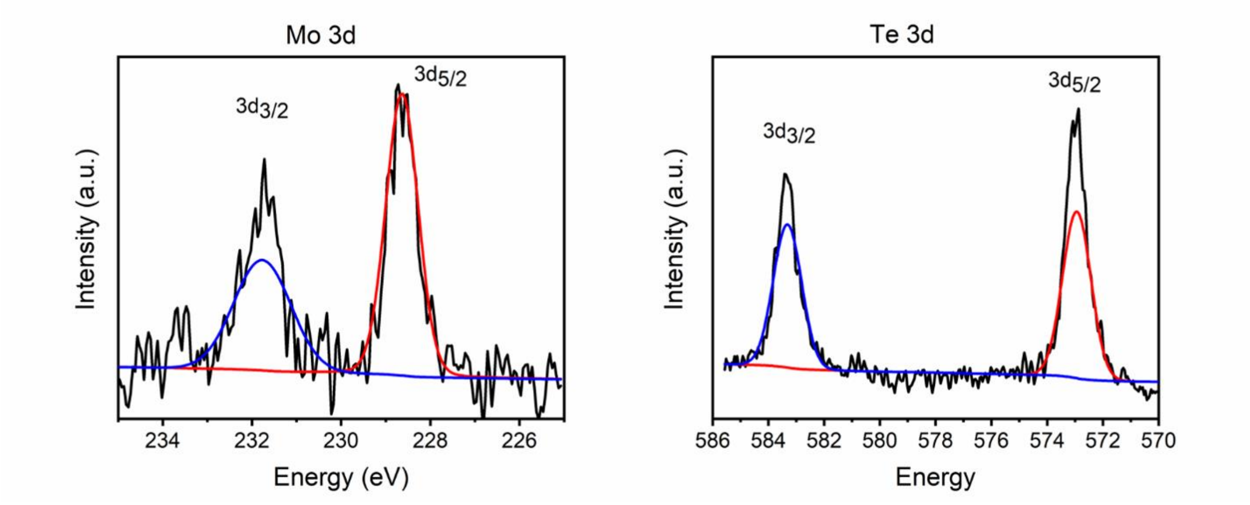

Supplementary Figure 2. XPS spectra of freshly exfoliated $MoTe_2$ flake. Mo binding energies at 228.64 eV and 231.77 eV correspond to $3d_{5/2}$ and $3d_{3/2}$ Mo orbitals, respectively. Te binding energies at 572.93 eV and 583.31 eV correspond to $3d_{5/2}$ and $3d_{3/2}$ Te orbitals, respectively. This is in good agreement with Te-Mo bonding.

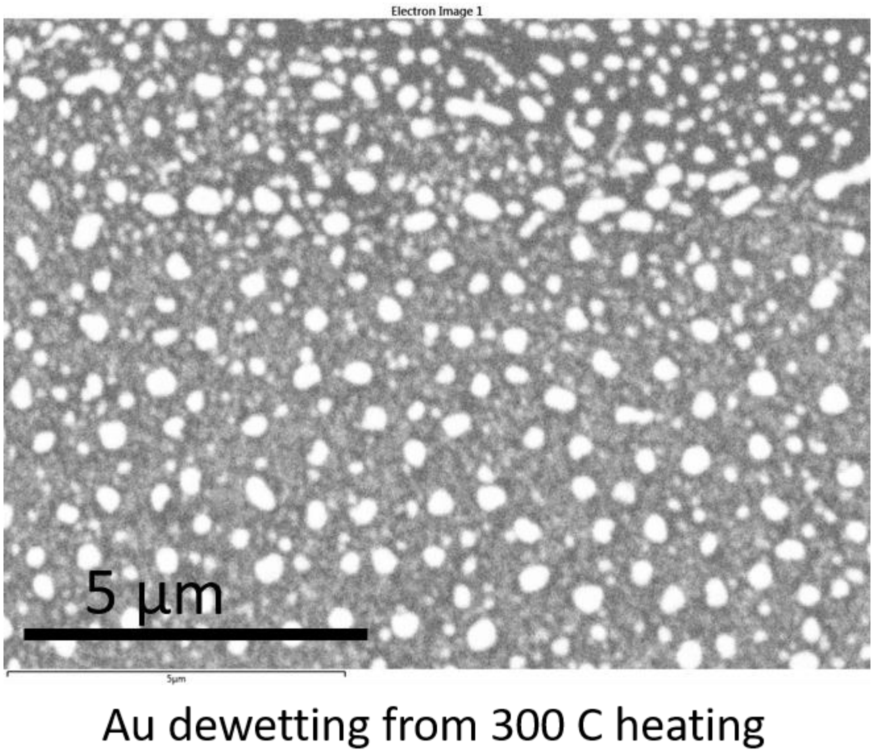

Supplementary Figure 3. SEM image of Ti/Au film after heating at 300 °C showing the formation of droplets due to dewetting.
